# Calcified cystic lesion in cerebellum: A case report

**DOI:** 10.1016/j.radcr.2024.03.061

**Published:** 2024-04-20

**Authors:** Hardita P. Yudhanto, Widiana Ferriastuti, Suresh K. Mukherji

**Affiliations:** a Department of Radiology, Faculty of Medicine Universitas Airlangga - Dr. Soetomo General Hospital, Surabaya, Indonesia; b Department of Radiology, College of Medicine, University of Illinois, Chicago, IL, USA

**Keywords:** Intracranial epidermoid cyst, Imaging, Hypodense, Calcified, Computed tomography scan, Magnetic resonance imaging, Restricted diffusion area

## Abstract

Intracranial epidermoid cysts are benign, slow-growing congenital tumors of ectodermal origin. They are rare embryonal benign cystic masses with an incidence rate of approximately 0.04%-0.6% of intracranial tumors. Computed tomography (CT) and magnetic resonance imaging (MRI) are fundamental diagnostic tools providing valuable information for surgical management. We reported a 59-year-old male patient with right limb weakness twelve hours prior to admission, slurred speech, and paresis of the facial nerve. Based on history taking, physical examination, and radiology examinations, we concluded a diagnosis of non-communicated hydrocephalus due to a right cerebellar intra-axial tumor with a suspicion of low-grade glioma (Pylocitic Astrocytoma). CT and MRI in intracranial epidermoid cysts are fundamental diagnostic tools for diagnosing and obtaining helpful information for surgical planning. Intracranial epidermoid cysts appear as lobulated lesions filling and expanding CSF spaces and exerting a gradual mass effect, insinuating between structures and encasing adjacent nerves and vessels. In this case, we noted a hypodense lesion with irregular calcifications and well-defined on the right cerebellar region measuring 6.15 × 5.47 × 5.7 cm, surrounded by a hypodense image suggesting an intra-axial mass suspected of low-grade glioma with a differential diagnosis of brain abscess. The hypointense lesion on the T1WI sequence found in the MRI examination, with no significant contrast enhancement and restricted diffusion area on DWI, was one of the notable features described in the epidermoid cyst. Intracranial epidermoid cyst rarely occurs in the intracranial, resulting in many symptoms in this case, which should be diagnosed and treated promptly. Imaging aids in proper diagnosis and provides more valuable information for further treatment.

## Introduction

Intracranial epidermoid cysts are sporadic benign congenital tumors arising from ectoderm, with an incidence rate of roughly 1% of all intracranial tumors [1]. With their slow-growing behaviour, patients are usually asymptomatic until 20-40 years of age. Nevertheless, they usually begin to manifest in the second to fourth decades of life. Intracranial epidermoid cysts are located supra-infratentorial, mainly in the cerebellopontine angle (CPA, 40%) and suprasellar cisterns (18%), less commonly in Sylvian fissure, cerebral and cerebellar hemispheres, and lateral and fourth ventricles [2]. They emerge as epithelial sequestrations from the third until the fifth week of development and persist after the closure of the neural tube. These intracranial epidermoid cysts grow due to the accumulation of desquamated cells and their breakdown products, such as keratin and cholesterol, with the growth rate linear to that of the epidermis [3]. These cysts are formed primarily in the sellar region, juxtasellar region, juxta-cavernous sinus, lateral fissures, brainstem, posterior cranial fossa, juxta-fourth ventricle, anterior cranial fossa bottom, spinal canal, and larger cysts grow across the cranial fossa [4].

Clinical presentations usually reported by the patients include headaches (most common), cranial nerve deficit, cerebellar symptoms, seizures, and raised intracranial pressure. Computed tomography (CT) and magnetic resonance imaging (MRI) hold a substantial role in depicting this entity. Intracranial epidermoid cysts appear as lobulated lesions that fill and expand CSF spaces and exert a gradual mass effect, insinuating between structures and encasing adjacent nerves and vessels [5]. Treatment of intracranial epidermoid cysts consists of simple aspiration and total or subtotal excision, depending on the adherence of the tumor capsule to the surrounding neurological and vascular structures [6].

## Case report

A 59-year-old man reported weakness of the right limb 12 hours prior to his presentation, with stiffness in the right hand and leg, slurred speech, and right facial paresis. He also described repeated headaches that had been occurring for 6 months and decreased hearing. Nonetheless, he denied ear and tooth infections and weight loss. A history of Parkinson's disease was recorded, and he had been taking Levodopa and Mecobalamin since 2016. On physical examination, we found paresis of the right seventh, eighth, and twelfth cranial nerves, along with right motoric lateralisation.

**Fig. 1:**
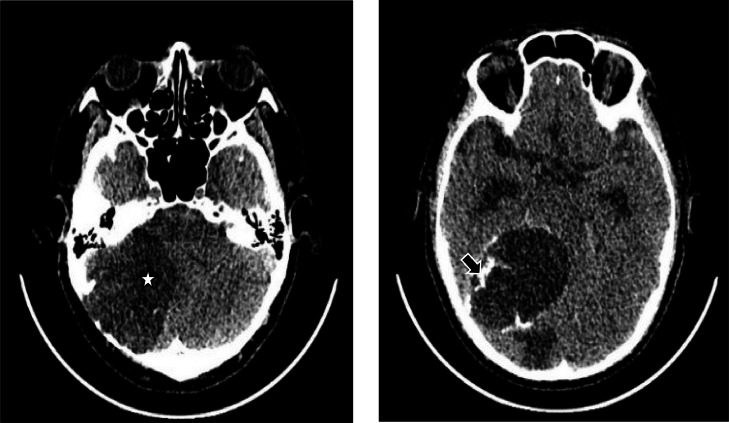
Unenhanced head MSCT shows a hypodense lesion with a clear border (star) containing irregular calcification (black arrow).

The heart and lungs appear normal in the chest radiograph. Unenhanced head CT revealed a hypodense lesion with irregular calcifications and a well-defined border on the right cerebellar region measuring 6.15 × 5.47 × 5.7 cm accompanied by a surrounding hypodense shadow, suggesting an intra axial mass, suspected of low-grade glioma with a differential diagnosis of brain abscess (Fig. 1); erosion in the right occipital bone; distorted fourth ventricle to the contralateral; bilateral lateral ventricles with FH/ID ratio of less than 0.5 and FH/BPD less than 0.3, temporal horn less than 2 mm; third ventricle ballooning, and distorted impression of noncommunicating hydrocephalus.

**Fig. 2:**
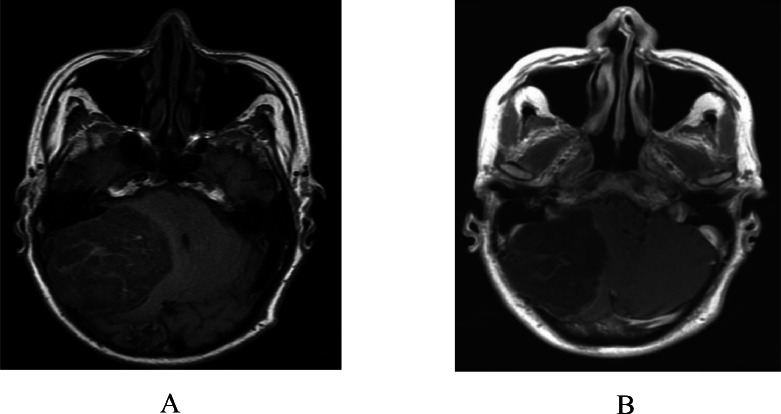
(A) Axial T1 MRI shows the isointense lesion, which, on contrast administration (B), shows subtle contrast enhancement.

**Fig. 3:**
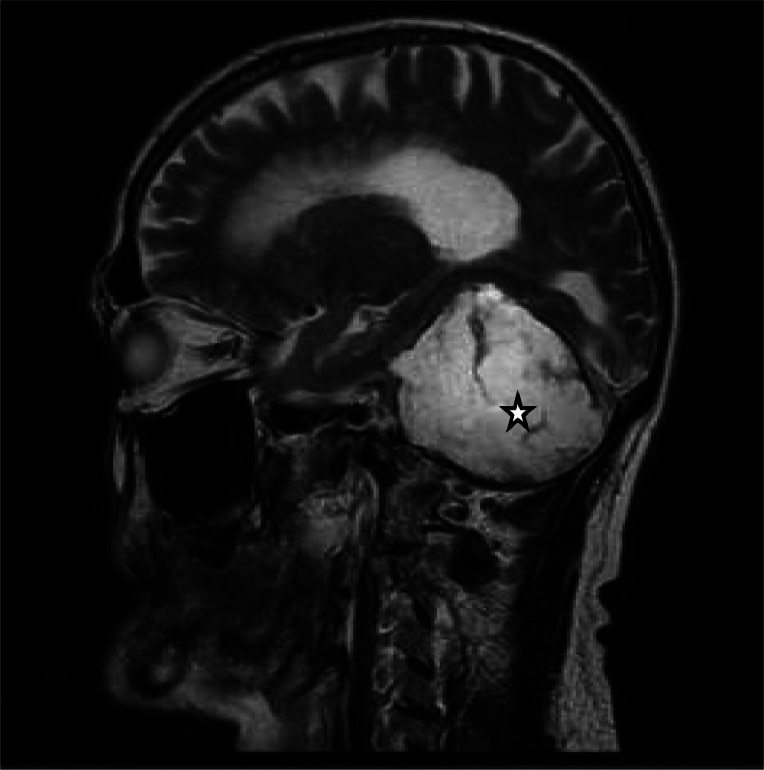
The sagittal view of T2 head MRI shows the hyperintense lesion (star sign).

**Fig. 4:**
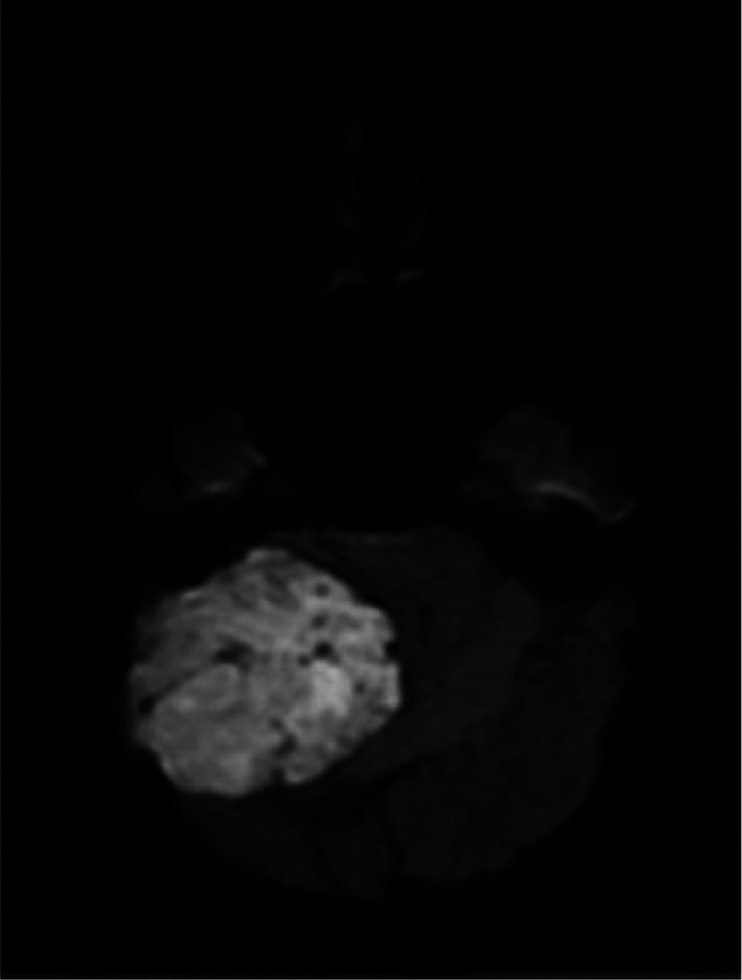
Restricted diffusion area on DWI.

Subsequent MRI with and without contrast depicted a cystic lesion, located intra-axially, with an irregular margin in the right cerebellar hemisphere, which might be an epidermoid cyst, along with noncommunicating hydrocephalus, subacute ischemic cerebral infarction in the left corona radiata, and small vessel ischemia in the right and left centrum semiovale, right and left cortical-subcortical frontoparietal lobes. The lesion appeared isointense on T1WI (Fig. 2A), hyperintense on T2WI (Fig. 3), and restricted diffusion area on diffusion-weighted imaging (DWI) (Fig. 4), which, on contrast administration, showed subtle contrast enhancement (Fig. 2B).

**Fig. 5:**
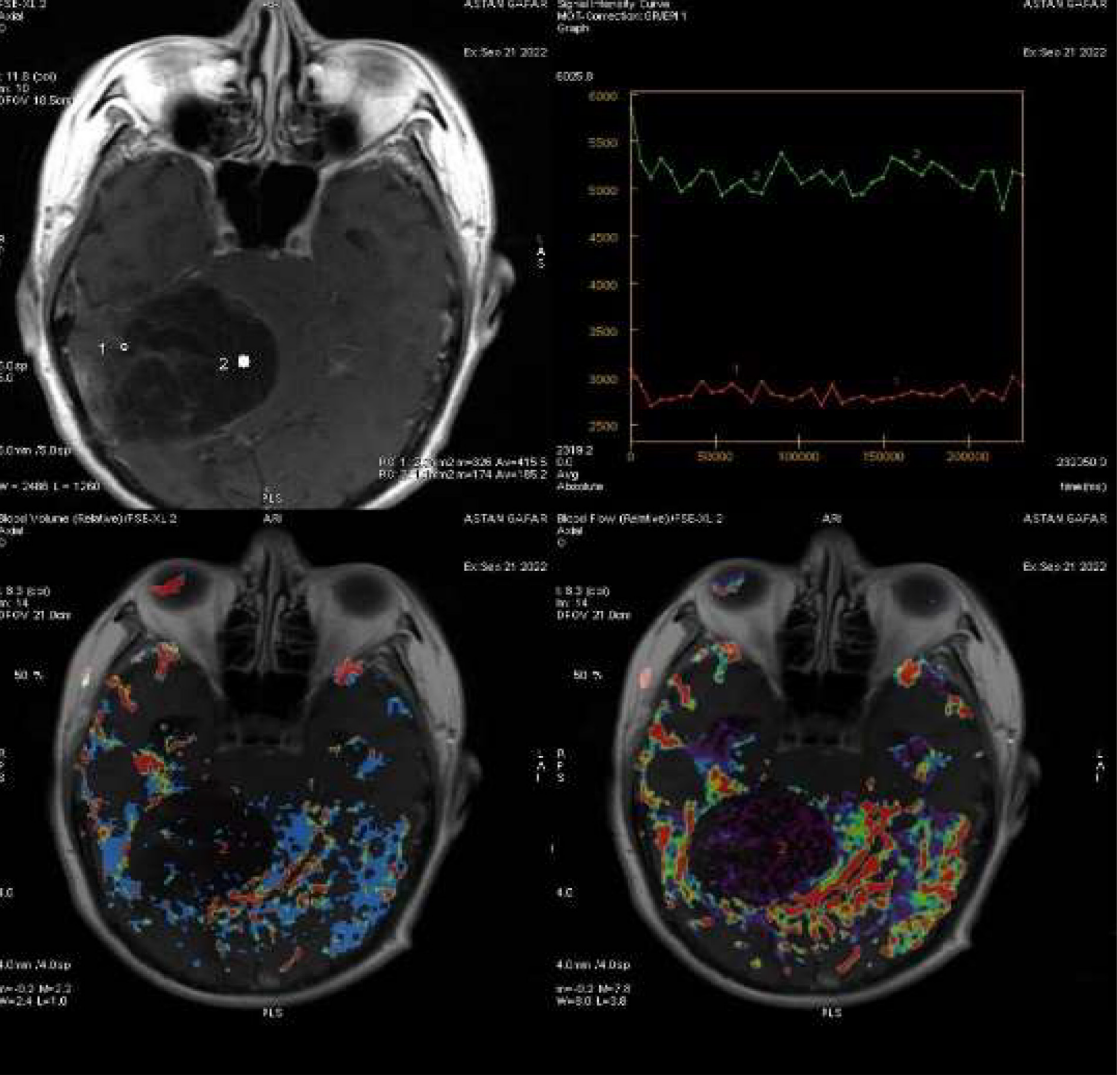
Head perfusion MRI.

**Fig. 6:**
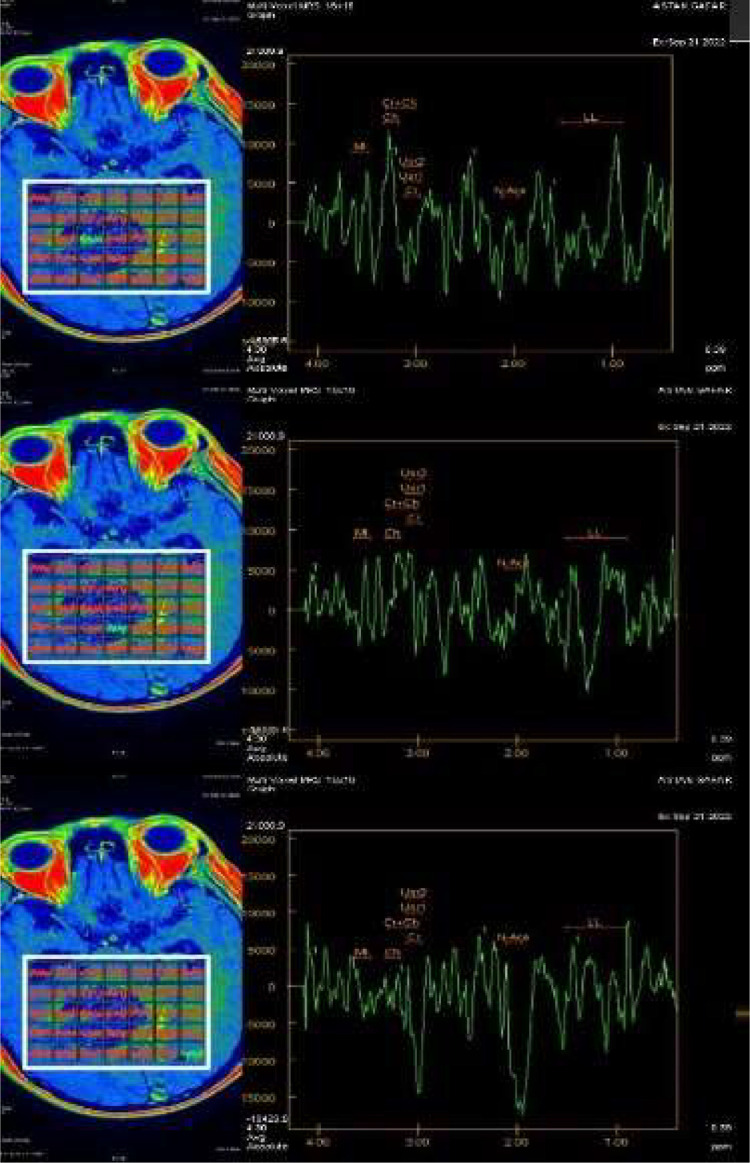
MR spectroscopy shows no increase in the Ch/Cr and Ch/NAA ratios.

MR angiography showed patent Circulus of Willis with no aneurysm or vascular malformation, with no increase in the ratio of Choline/Creatinine and Ch/NAA on MR Spectroscopy (Fig. 6) and no increase in rCBV on MR perfusion (Fig. 5).

**Fig. 7:**
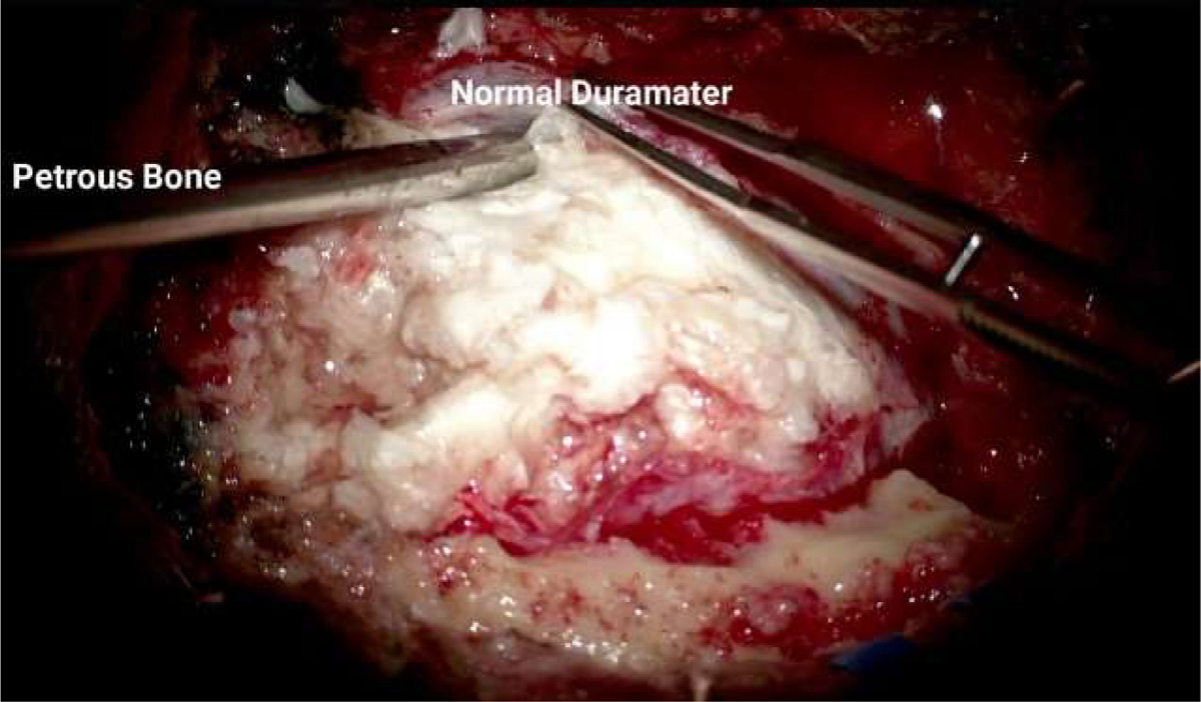
The epidermoid cyst was revealed during the surgery.

**Fig. 8:**
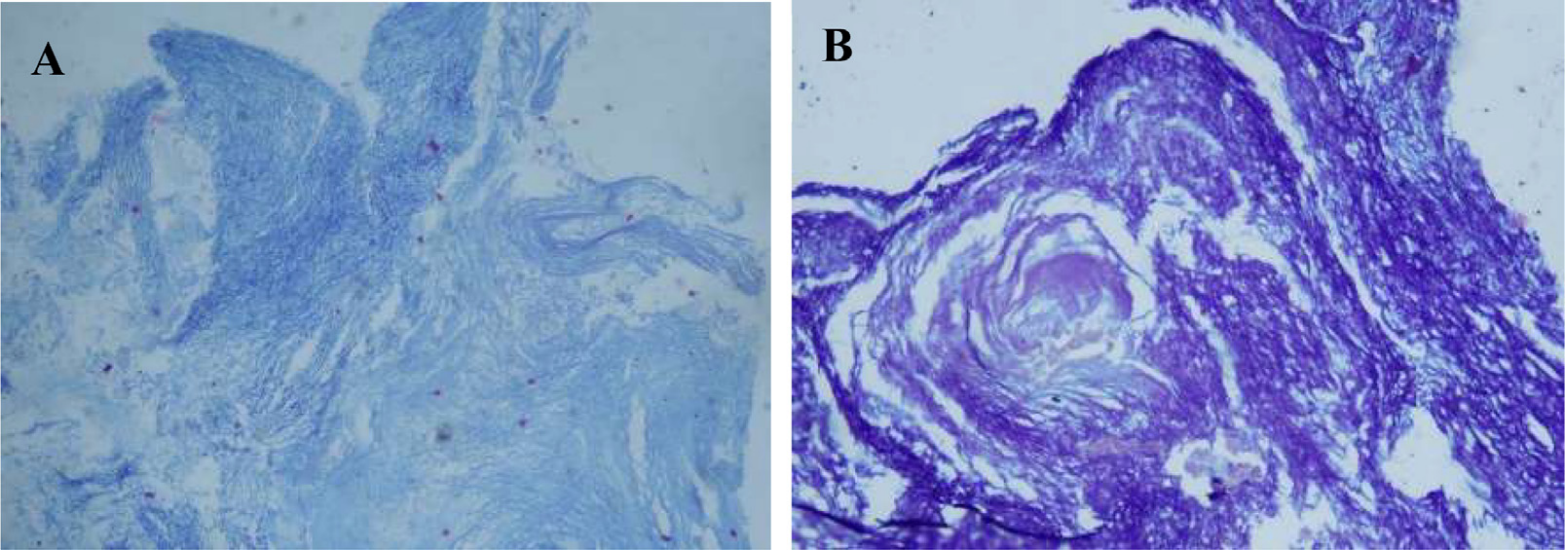
Pathology examination of the cyst using Ziehl Nelseen stain shows extensive squamous tissue with no epithelial findings nor signs of malignancy. (A) 20x magnification and (B) 40x magnification.

A craniotomy for tumor excision was ordered and yielded a satisfactory result with no complications. The obtained tumor tissue was then sent to the pathology and microbiology laboratorium for analysis (Fig. 8), which led to the postoperative diagnosis of a right intra-axial cerebellar tumor suspected of epidermoid cyst and noncommunicating hydrocephalus (Fig. 7). The findings confirmed that the central ``islands'' within the lesion were composed of keratin debris and cholesterol components.

## Discussion

According to the literature, the intracranial epidermoid cyst is a rare embryonal benign cystic mass and accounts for approximately 0.04%-0.6% of intracranial tumors [7]. Epidermoid cysts are epithelial cells remaining in the neural crest ectoderm during the third to fifth week of embryonic development and wrapped with the closure of neural tubes. Eventually, the cysts are formed in the sellar and juxta sellar region, juxta cavernous sinus, lateral fissures, brainstem, posterior cranial fossa, juxta-fourth ventricle, base of anterior cranial fossa, and spinal canal, whereas larger cysts grow across the cranial fossa [8]. Intracranial epidermoid cysts are uncommon congenital lesions and usually very slow-growing; thus, their clinical manifestation might be delayed for many years. Typically, patients begin to experience symptoms during the third to fourth decade of their life. The prevalence seems to be increased in male patients, although it might not be found in all series [5]. However, with advancing age, hormonal changes lead to increased secretion of glands, resulting in the rapid growth of tumors and spontaneous ruptures, and subsequently stabilise during adulthood due to steady hormonal levels, while dermoid cyst is less affected by the hormone and might cease to grow [9]. A large body of evidence has described the signs and symptoms of intracranial epidermoid cyst, including headache, cranial nerve paresis, cerebellar symptoms, seizures, and elevated intracranial pressure [5], which were pronounced well in our patient.

CT and MRI are fundamental diagnostic tools that diagnose and provide essential data for surgical planning. An epidermoid cyst is presented on a non-enhanced CT scan as a well-defined, lobulated, low-density mass insinuating around the cisterns, sometimes with deep invagination into the adjacent brain parenchyma. Most epidermoid cysts are non-enhancing but may show calcification or hemorrhage. Moreover, a CT scan allows the study of diploic erosion, which is unusual to observe; however, a massive diploic erosion can be discerned in giant epidermoid cysts. The combination of cellular debris along with a high cholesterol content may lower the density of the epidermoid to approximately 0 HU and can thus be identical to the density of CSF and look similar to an arachnoid cyst. Calcification is seen in a minority of cases (10%-25%), as in our patient [8], and rarely, an epidermoid cyst may be hyperdense due to hemorrhage, saponification, or high protein content (“white epidermoid”).

MRI allows for better characterisation of the lesion, showing low-intensity signals on T1-weighted images, high-intensity on T2-weighted images, and heterogeneous signals on FLAIR due to cholesterol and cellular debris, with moderate peripheric enhancement and diffuse hypersignal. Epidermoid cysts show restricted diffusion with higher signal intensity than that of cerebrospinal fluid (CSF) on diffusion-weighted imaging, which is also described well in our case. Bone erosion accompanying the epidermoid cyst appears to result from traumatic embedding of squamous epithelium in the bone [5,6]. The cyst was lined by fibro-inflammatory tissue composed of multinucleated giant cells and haemosiderin-laden macrophages. Prominent foci of dystrophic calcification were present, particularly along the fibrous cyst wall, with patchy chronic inflammation. The intra-cystic component was comprised of central keratin debris and cholesterol clefts [10]. Eventually, the above characteristics were presented well in our radiologic findings.

## Conclusion

This manuscript described a rare case of intracranial epidermoid cyst, which should be diagnosed and treated promptly. Our case described facial paresis and decreased hearing, which was reported by the patient. We also specified the importance of CT scans and MRI in evaluating the tumor, proposing the significance of restricted diffusion area on DWI and subtle contrast enhancement upon contrast administration. Nonetheless, biopsy remains the gold standard modality and provides more valuable information for further treatment, such as surgery. Furthermore, apart from being used as a diagnostic tool, radiology could contribute as a predictor of serious complications.

## Patient consent

Written consent was obtained from the patient, as no identifiable patient data was included in this case report. This study met the ethical principles and received approval from our hospital's Research Ethics Committee.
